# Upraising *Stenotrophomonas maltophilia* in Critically Ill Patients: A New Enemy?

**DOI:** 10.3390/diagnostics13061106

**Published:** 2023-03-15

**Authors:** George Dimopoulos, José Garnacho-Montero, Elisabeth Paramythiotou, Antonio Gutierrez-Pizarraya, Charalambos Gogos, Maria Adriansen-Pérez, Chrysa Diakaki, Dimitrios K. Matthaiou, Garyphalia Poulakou, Karolina Akinosoglou

**Affiliations:** 1 Department of Critical Care, Evgenidio Hospital, Medical School, National and Kapodistrian University of Athens, 157 72 Athens, Greece; 2 Unidad Clínica de Cuidados Intensivos, Hospital Universitario Virgen Macarena, 41009 Sevilla, Spain; 3 Department of Critical Care, University Hospital ATTIKON, 124 62 Athens, Greece; 4 Department of Internal Medicine, University General Hospital of Patras, Medical School, University of Patras, 265 04 Patras, Greece; 5 Unidad Clínica de Cuidados Intensivos, Hospital Universitario Virgen del Rocío, 41013 Sevilla, Spain; 6 Department of Critical Care, Athens Hospital, 112 57 Athens, Greece; 7 3rd Department of Internal Medicine, SOTIRIA Hospital, Medical School, National and Kapodistrian University of Athens, 115 27 Athens, Greece

**Keywords:** *Stenotrophomonas maltophilia*, carbapenem resistance, cotrimoxazole, ventilator-associated pneumonia, emerging pathogens, intensive care unit

## Abstract

*Stenotrophomonas maltophilia* (*S. maltophilia*), an important pathogen in immuno-compromised patients, has recently gained attention in patients admitted in intensive care units (ICU). We sought to investigate clinical features of infections caused by *S. maltophilia* in ICU patients and identify risk factors for mortality. We conducted a retrospective study in two multivalent non-COVID-19 ICUs of tertiary-teaching hospitals in Greece and Spain, including patients with isolated *S. maltophilia* from at least one clinical specimen along with clinical signs of infection. A total of 103 patients (66% male) were analyzed. Median age was 65.5 (54–73.3) years and mean APACHE II and SOFA scores upon ICU admission were 18.36 (±7.22) and 18.17 (±6.95), respectively. Pneumonia was the predominant clinical syndrome (72.8%), while 22% of cases were among hemato/oncology patients. Crude 28-day mortality rate was 54.8%, even though, 14-day clinical and microbiological response was 96%. Age, APACHE II on ICU admission, hemato-oncologic disease, and multi-organ failure were initially identified as potential predictors of mortality. In the multivariable analysis, only increasing age and hemato-oncologic disease were shown to be independent risk factors for 28-day mortality. High all-cause mortality was observed in critically ill patients with predominantly respiratory infections by *S. maltophilia*, despite initial clinical and laboratory response after targeted treatment. The study elucidates a potentially worrisome emerging pathogen in the ICU.

## 1. Introduction

*Stenotrophomonas maltophilia* (*S. maltophilia*) is a ubiquitous Gram-negative non-glucose fermenter, previously known as *Xanthomonas maltophilia* or *Pseudomonas maltophilia*. Although known as a low-virulence microorganism, *S. maltophilia* has been recognized as an important pathogen in specific populations, particularly hemato-oncologic patients undergoing chemotherapy [1,2,3,4]. Infections in these populations have been associated with high rates of mortality, partly attributed to the multidrug-resistant profile of this pathogen, either as healthcare-acquired or within the rapidly evolving community-acquired resistance [1,2,3,4,5].

In recent years, an increased recovery of *S. maltophilia* was documented from multiple specimens of critically ill, ICU-admitted patients and neonates, being identified as the cause of primary or secondary bacteremia, catheter-related infections, nosocomial pneumonia, surgical infections, endocarditis, skin and soft tissue infections, etc. [3,6]. Even though commonly isolated due to secondary bacteremia, primary bacteremia is not uncommonly reported, recorded in 45% of patients in a recent study [7,8,9]. The increasing incidence of *S. maltophilia* infections has been highlighted during the COVID-19 epidemic [10]. Recent studies have shown increased prevalence of *S. maltophilia* infections, among hospitalized COVID-19 patients, leading the race of pathogens together with *A.baumani* [11]. *S. maltophilia* has also been recognized as a cause of outbreaks and pseudo-outbreaks, the latter being attributed to the colonization of bronchoscopic and other respiratory equipment by this microorganism [12,13,14]. The latest data from Greece reviewing 68 cases of *S. maltophilia* over a 5-year period, almost equally divided among medical, surgical, hematology/oncology, and ICU departments, showed a predominance of respiratory tract (54.4%), bloodstream (16.2%), skin/soft tissue (10.3%), and intra-abdominal (8.8%) infection [15]. Similarly, previous multicenter data from Spain [16] reported a variable incidence of 3.4 to 12.1 per 10,000 admissions predominantly in ICU (32%) and surgical patients (18%). Respiratory tract (46%) and surgical site infections were the main sites of *S. maltophilia* isolation (14%).

However, data in both settings remain scarce and relatively old, extending over a decade ago, not representing current antimicrobial susceptibilities and trends. Interestingly, even though both studies [15,16] showed increased susceptibility to quinolones and trimpethoprin/sulfomethoxazole ensuring best outcomes, the rising resistance of *S. maltophilia* in both regimens during the last years is concerning [17]. Of note, current guidance is provided as “suggested approaches” based on expert opinion, clinical experience, and available literature often supporting combination treatment [18], while current and endemic epidemiology—which is largely unknown—should be taken into account [19].

In this setting of scarce data, we aimed to investigate the current clinical profile of ICU patients in whom *S. maltophilia* is recovered from clinical samples and identify risk factors for mortality.

## 2. Materials and Methods

A retrospective study was conducted in the multivalent non-COVID-19 ICU of two tertiary teaching hospitals in Greece and Spain: Attikon University Hospital with an 18-bed polyvalent ICU (Athens, Greece) and Hospital Virgen del Rocío, a 62-bed polyvalent unit in a large urban hospital with teaching accreditation (Seville, Spain). The second hospital has a solid organ transplantation (liver, heart, kidney) and an active bone marrow transplantation program. This study complies with the Declaration of Helsinki and Good Clinical Research Practice principles. The Institutional Review Board of both hospitals approved the study; the need for informed written consent was waived because of the observational nature of the study and handling of patient information according to general data protection regulations.

Patients were included in the study if they had been hospitalized during the period 2020–2021 and provided that they yielded *S. maltophilia* from at least one clinical specimen along with clinical signs of infection and a decision of the treating physician to treat for *S. maltophilia* upon identification of the pathogen. The following demographic and clinical data were collected: age, gender, underlying disease(s), antimicrobial therapy prior to and after *S. maltophilia* isolation, ICU length of stay, hospital stay. Outcomes were assessed including 28-day mortality (primary endpoint), ICU and hospital mortality, and 14-day clinical and microbiological response. A positive clinical and microbiological response was assessed on day 14 and defined as resolution of signs and symptoms of infection, sterilization of blood cultures within 7 days of treatment initiation, and absence of recurrent infections. Patients’ severity and organ dysfunction were estimated by the Acute Physiology and Chronic Health Evaluation (APACHE) II score and the Sequential Organ Failure Assessment (SOFA) score, both calculated on the day of ICU admission and on the day a culture positive for SM was sent [20,21].

### 2.1. Definitions

Source of infection was assigned according to Centers for Disease Control and Prevention definitions [22]; presence of severe sepsis or septic shock on the index date was based on standard definitions [23,24]. Hospital-acquired pneumonia (HAP) was defined according to the American Thoracic Society 2005 and 2016 definitions, whereas Ventilator-Associated Pneumonia (VAP) was defined as pneumonia occurring 48–72 h after ICU admission [25]. Further diagnostic criteria included a quantitative culture of endotracheal aspirate revealing ≥10^5^ cfu/mL; development of new or persistent infiltrates in the chest X-ray; presence of fever (temperature < 36 °C or >38.5 °C); white cell count >11,000/mL or <4000/mL; declining ratio of partial pressure to inspired fraction of oxygen (PaO_2_/FiO_2_ ratio) [25,26]. Only the first episode of isolation of SM was studied. The treatment was considered as adequate when the isolate was susceptible to at least one of the administered antimicrobials dosed appropriately.

### 2.2. Microbiology

Each hospital conducted antibiotic susceptibility testing according to its own protocols. We report susceptibility as it was interpreted by the local laboratories. For blood culturing, the Vitek 2 automated system was used (bioMérieux, Marcy l’Etoile, France). Susceptibility was studied using automated systems or disk diffusion method and interpreted using 2021 Clinical and Laboratory Standards Institute (CLSI) breakpoints [27].

### 2.3. Statistical Analysis

Patients were divided into survivors and non-survivors. Categorical data are presented as group percentages and frequencies. Data normality was assessed using the Kolmogorov–Smirnov and Shapiro–Wilk tests using an a of 0.05. Continuous data with skewed distribution are presented as medians (first to third quartiles), respectively. Two-sample *t*-test and Fisher exact test were used for comparison of normally distributed continuous and categorical data, respectively. The Mann–Whitney U test was used for comparison of skewed continuous data, and Kruskal–Wallis was used to detect differences in non-normally distributed data. Bonferroni post hoc correction was used to assess multiple comparisons across groups. To explore associations between various patients’ characteristics and mortality, a multiple linear regression model in a backward elimination fashion (entry 0.05, removal 0.1) was performed. Goodness of fit was assessed with Hosmer–Lemeshow test. All tests were two-tailed, and statistical significance was considered for *p*-values < 0.05. Analyses were performed using SPSS for Windows (version 24.0 SPSS IBM Inc., Chicago, IL, USA).

## 3. Results

### 3.1. Clinical Attributes, Outcomes and Risk Factors for Non-Survival

A total of 103 patients, 68 of whom were male (66%) and had a median age of 65.5 years, were included in this study. Demographics and clinical characteristics of the patients are shown in Table 1; results are plotted as a total of patients and 28-day survivors/non-survivors. The main characteristics relating to the infection caused by *S. maltophilia* are shown in Table 2. Non-survivors were found to be significantly older and have a higher APACHE II score upon ICU admission (*p* = 0.01 and *p* = 0.032, respectively) (Table 1). Hematologic/oncologic patients were more likely to die (*p* = 0.004) (Table 1). Relating to the infection caused by *S. maltophilia*, the majority of patients were suffering from sepsis, while respiratory tract infection was the predominant site of inflammation that the pathogen was also isolated, followed by surgical site infections and central catheters (Table 2). A significant proportion of patients had been priorly exposed to carbapenems or piperacillin/tazobactam, whereas a co-trimoxazole or levofloxacin-based regimen was administered to deal with *S. maltophilia* infection. No significant differences were shown between survivors and non-survivors in the reported parameters (Table 2). Patients with respiratory tract infection (*n* = 75) due to *S. maltophilia* presented 14-day clinical and microbiological response in 96% and 96% of cases, respectively. However, hospital mortality rates for these patients reached 53%, and 28-day mortality reached 54.7%. Rates were higher among hemato/oncologic patients. Even though 14-day clinical and microbiological response reached 100%, within hospital and 28-day mortality was reported to be 77.3 and 81.8, respectively. Notably, mortality in hemato/oncologic patients significantly differed from patients with non-hematologic/oncologic disease (*p* = 0.015 for hospital mortality and 0.004 for 28-day mortality). Following adjustment for significant variables in the univariate analysis, namely age, APACHE II score upon ICU admission, multi-organ failure, and hemato-oncologic disease, only age and hemato/oncologic disease were found to be independent predictors for mortality (Table 3).

### 3.2. Microbiology and Antimicrobial Treatment

All 103 patients were on antibiotics when *S. maltophilia* was isolated. Forty-one patients (39.8%) were exposed to carbapenems, either alone (17 patients; 16.5%) or as a combination with a glycopeptide or linezolid. Fifty-two patients (50.5%) received β-lactams either alone, (27 patients, 26.3% piperacillin /tazobactam as a single agent) or in various combinations with a quinolone and/or an anti-Gram-positive agent. Finally, six patients (5.8%) were receiving only a glycopeptide or linezolid. No patient was receiving cotrimoxazole prior to *S. maltophilia* isolation. Upon identification of *S. maltophilia*, 57 patients received cotrimoxazole as an add-on strategy; in three of them, an escalation of the backbone was performed either with vancomycin (two patients) or with meropenem (one patient). Two patients were already on levofloxacin; therefore, two active drugs against *S. maltophilia* were administered. Among 46 patients who received levofloxacin-based combinations as an add-on strategy, three isolates exhibited intermediate susceptibility to levofloxacin and in 10 additional isolates, and the susceptibility to levofloxacin was not reported. Isolates exhibited 97.2% susceptibility to cotrimoxazole, 93.2% susceptibility to minocycline, and 59.2% susceptibility to levofloxacin. In one patient, the isolate was reported as only SXT susceptible. Other antibiotics such as colistin, ceftazidime, ciprofloxacin, tigecycline, and ticarcillin were reported very scarcely in the antibiograms to allow for solid conclusions. Among three patients with a *S. maltophilia* isolate non-susceptible to cotrimoxazole, one was administered cotrimoxazole with successful clinical and laboratory response on day 14, despite the lack of in vitro susceptibility. All four patients receiving inadequate treatment responded clinically and microbiologically on day 14; however, 50% 28-day mortality was observed.

### 3.3. Other Pathogens and Coinfections

In 37 out of 103 patients (35.9%), a second microorganism was recovered from the same specimen; in only 15 of them, it was considered a co-infection (*A. baumannii* five cases, Enterobacteriaceae eight cases, *P. aeruginosa* one case, *E. faecalis* one case). In one additional case, co-infection was attributed to a pathogen originating from another specimen/infectious site. On the other hand, 23 species were recovered from the same specimen that were deemed as colonizers including: 11 fungi (7 *C. albicans*, 4 *Aspergillus* spp.), 4 Enterobacteriaceae, 4 rare skin or water commensals (*Leclercia adecarboxylata* and *Corynebacterium* sp.), and 4 enterococci. The presence of coinfection portended 50% mortality (ICU, 28-day, and hospital mortality). However, the difference compared to patients without coinfection was not significant. The presence of coinfection was associated with significantly worse clinical and laboratory response (2/14 patients) on day 14, compared to counterparts without coinfections (1/87 patients), denoting 14.3% versus 1.1% clinical and microbiological failure rates, respectively (*p* = 0.013 for both comparisons).

## 4. Discussion

This retrospective multicenter study represents a contemporary report of ICU patients with *S. maltophilia* infection and includes one of the largest case-series of respiratory infections in this population. This fact enabled us to upraise epidemiology of *S. maltophilia* infections in the multivalent ICU setting, describe one of the highest rates of crude mortality of 54.8% in this specific population, and investigate the particular characteristics of subgroups of patients. High all-cause mortality approaching and even exceeding that reported in hemato-oncologic cohorts is an important conclusion from this study, despite the initial clinical and laboratory response after adapted treatment [28].

Well-recognized risk factors for emergence of *S. maltophilia* infections (such as hematologic or oncologic malignancy and solid organ or bone marrow transplantation) accounted for about 21.3% of the population in this study. It is important to note that a considerable proportion of 27% of patients did not have any comorbidity, and an equally important 24.3% had only cardiovascular disease, along with dyslipidemia/obesity and hypertension in a small percentage of cases, which are factors not known to confer vulnerability to infections by low virulence pathogens such as *S. maltophilia*. COPD has been recognized in previous studies as a risk factor for the emergence of *S. maltophilia* infections in ICU patients [29]. Both previous Spanish and Greek cohorts identified presence of COPD in 25% and 30.9%, respectively [15,16]. Almost 9% of our patients had underlying chronic respiratory infections (mostly COPD), probably indicating increased consumption of antibiotics and/or corticosteroids. This comes in line with data from a recent meta-analysis including 2320 cases of *S. maltophilia* infection in the ICU, which showed that severe disease, as this reflected in APACHE-II score > 20, was the most important risk factor for ICU acquired *S. maltophilia* infection [30]. COPD (OR = 3.97), malignant tumor (OR = 2.15), mechanical ventilation (OR = 8.75), tracheotomy (OR = 6.12), endotracheal intubation (OR = 4.25), β- Lactamase inhibitors (OR = 9.98), aminoglycosides (OR = 4.01), carbapenems (OR = 2.82), and quinolones (OR = 2.17) posed significant risk on the acquisition of *S. maltophilia* pneumonia [30]. It appears that patients at risk for *S. maltophilia* infection are highly variable, leading to further questions as to their best management [31].

An important feature of this study is the predominance of pneumonia as a clinical syndrome across all subgroups of patients (medical, surgical, and haematological patient). This comes as no surprise and has been previously recorded both in a Greek and Spanish cohort [15,16]. It appears that the total duration of the artificial airway and ventilator use, gastric tube placement, acid suppressant, and antibiotics (especially carbapenem) play a role in the incidence of *S. maltophilia* associated HAP in severe, long-stay ICU patients [32,33]. Despite the large body of the literature, we did not observe many bacteremic infections by *S. maltophilia*; a probable explanation would be the population served by the participating centers, which were not dedicated to hemato-oncology specialties [28,34].

Exposure to a carbapenem is common for ICU patients and represents a well-established risk factor for the recovery of *S. maltophilia* [15,16]. This was not very prevalent in our study, since only 40% of the studied population had recent exposure to carbapenems. Carbapenem resistance has dramatically increased in hemato-oncology patients with gram negative bacilli BSI in recent years and is associated with a worse outcome, especially for non-fermenting bacteria including *Acinetobacter* and *Stenotrophomonas* [35]. In a real-world, multicenter, retrospective case-control study from five centers in the southeast United States assessing 325 patients, *S. maltophilia* non-susceptibility had a prevalence of ∼50% to at least one first-line or commonly used agent [36]. A recent report from the UK showed that among patients colonized/infected with carbapenem non-sensitive gram-negative bacteria, 29% were *S. maltophilia* [37]. At the same time, analysis of co-infections showed that 50%–85% of patients with carbapenem-resistant gram-negative bacilli bloodstream infections (BSI) had also pulmonary infections. Sputum culture results suggested that sputum culture positivity rate was as high as 57.1%–66.7% in patients with carbapenem-resistant *A.baumannii* and *S. maltophilia* BSI [35]. The latter underlies the need for infections control bundle implementation against HAP/VAP [38], but also the need for early screening of the respiratory tract specimens, in order to timely detect multidrug-resistant pathogen colonization and protect patients from breakthrough BSI.

In contrast with other authors, we were not able to demonstrate any detrimental effect of co-infections on survival. Co-infections adversely impact 14-day clinical and microbiological response. Presence of co-infection with *S. maltophilia*, viruses, or bacteria shows a potential synergistic effect in mortality as in the case of *Pseudomonas* or COVID-19 in critically ill patients [39,40]. We also did not observe frequent co-infection with enterococci, probably because a vast majority of our patients did not have chemotherapy-induced gut mucositis [4,7]. At this point, someone could debate the true significance of *S. maltophilia* from respiratory samples. Indeed, clear distinction of infection from colonization is very difficult. *S. maltophilia* represents a colonizing organism or a true pathogen, particularly in patients with underlying pulmonary conditions such as cystic fibrosis or ventilator dependency, and especially in the setting of polymicrobial infections [7,41,42,43]. However, in our cohort, the rate of recovery of co-pathogens from the same sample was low, indicating that *S. maltophilia* in the context of a well-described clinical syndrome (associated in the majority of our patients with expression of severe sepsis or septic shock) would represent rather a true pathogen than a colonizer. Furthermore, most of our patients were administered as an add-on strategy a single antibiotic active against *S. maltophilia* while cases of empiric escalation of the whole regimen were rare (three patients). This fact precludes treatment of other potential pathogens on an empirical basis and confirms the belief of the treating physician in the recovery of a “true pathogen” from respiratory samples. In a recent report of 33 patients with respiratory infections and documented difficulty to distinguish between colonization and infection on the clinical grounds, the authors suggested that mechanical ventilation, prolonged ICU stay, COPD, and underlying immunosuppression should prompt initiation of treatment in patients with recovery of *S. maltophilia* from respiratory infections [44]. Nonetheless, the clear role of *S. maltophilia,* however, as either colonizer or true pathogen, has been a matter of debate in non-immunocompromised populations, with clonality studies providing important clarifications.

However, selection of treatment regimen can prove problematic due to the impressive number of antimicrobial resistance genes and gene mutations carried by *S. maltophilia* isolates but also the accumulation of multidrug efflux pumps [45,46,47]. A “standard of care” antibiotic regimen for *S. maltophilia* infections against which to compare the effectiveness of other various therapeutic regimens does not exist, while recent data have shown no difference in mortality between currently selected treatment regimens including quinolones and co-trimoxazole [48]. On top of that, *S. maltophilia* antibiotic susceptibility testing and MIC threshold remains problematic [19].

The emergence of *S. maltophilia* has been associated with antibiotic overuse and misuse; especially carbapenem use [49], since this non-fermenter exhibits intrinsic carbapenem resistance [50,51]. Several reports showed increased recovery of *S. maltophilia* in parallel with *Acinetobacter baumannii* and other non-glucose fermenters, reflecting selection by antibiotic pressure [29,52]. We were not able to demonstrate any effect of carbapenems’ use in the patient’s outcome [50,53]. Cotrimoxazole was the most active in vitro antimicrobial in the studied population (97.1% susceptibility) and represented physicians’ most frequent choice, followed by levofloxacin, exhibiting a susceptibility of 59.2%. Susceptibility rates of cotrimoxazole and minocycline in this multicenter study were very high, despite growing evidence of emerging cotrimoxazole resistance among *S. maltophilia* strains [54,55]. High rates of susceptibility to cotrimoxazole probably did not allow us to detect any effect of inadequate treatment on mortality, as did other authors [3,4]. Fihmann et al. showed that inadequate treatment was associated with increased mortality (37.5%) in *S. maltophilia* infections compared to other non-fermenters, due to the multidrug resistant profile of this pathogen [3].

However, *S. maltophilia,* similarly to *A. baumannii,* represents a low-virulence pathogen; hence, the question is posed as to whether the patient finally dies due to its underlying disease and comorbidities rather than the *S. maltophilia* infection itself [4,29]. This comes in agreement with a study reviewing mortality from bacteremic *S. maltophilia* infections, where actually underlying conditions were mainly incriminated [56]. Age, APACHE II on ICU admission, hemato-oncologic disease, and multi-organ failure were potential predictors of mortality in the univariate analysis. Our data agree with a cohort analysis from hospitalized patients from the US, indicating that severity of disease, as reflected in severity score and multiorgan failure, including presence of respiratory or renal failure, as well as increased age and presence of comorbidities, worsen prognosis [57]. In the multivariate analysis, however, only increasing age and hemato-oncologic disease were elucidated as independent risk factors for 28-day mortality in our study. For this reason, a risk score for acquisition of *S. maltophilia* BSI in the hematological malignancy population was recently developed, so that patients who may benefit from early treatment are timely identified [58].

In our study, crude mortality was 54.3%, but four patients who were administered inappropriate treatment had a successful 14-day clinical and microbiological outcome. This is much higher than the previously recorded mortality of *S. maltophilia* infection barely reaching 4.4% in the Greek cohort. This is mainly attributed to the fact that, in our study patients exclusively derived from ICU and not from other departments as analyzed by previous authors. Vartivarian et al. have also reported mortality exceeding 50% in respiratory infections from *S. maltophilia* similar to our cohort [59]. Araoka et al. showed a 51% mortality in severely immunocompromised hemato-oncologic population with bacteremic *S. maltophilia* infections, elucidating neutropenia, damaged intestinal tract mucosa, and coinfection with enterococci as important features affecting mortality [7]. In a retrospective study from China of 51 cases of *S. maltophilia* bacteremia, mortality rate was 37.3%, while APACHE II was the only independent factor for mortality [60]. Similarly, data from Turkey showed that the 14th and the 30th-day mortality rates were 32.9% and 45.7%, respectively [61], while a Danish cohort revealed a 90-day mortality of 18% [62].

On the other hand, Barchitta et al. showed that exposure to surgical procedures prior to ICU admission increased nearly five-fold the risk of infection by *S. maltophilia* and highlighted mechanical ventilation as a risk factor [52]; Hanes et al. showed that trauma patients were prone to *S. maltophilia* late VAP, and lung contusion was an independent risk factor [4]. These studies provide a plausible explanation of the predominance of pneumonia in our subgroup of surgical admissions.

Our study has some limitations: (a) it is a retrospective study conducted in two different centers from two different countries, none of them serving a specific immunocompromised population; hence, even though among large series, the sample size is smaller than other ICU cohorts; (b) differences in laboratory practices did not permit us to collect data for a full range of antibiotic susceptibilities, enhanced by the fact that 97% of strains exhibited susceptibility to cotrimoxazole; and (c) we were not able to retrieve timing of adequate antimicrobial treatment. However, the strength of the study is the compilation of an adequate number of ICU patients with infections by *S. maltophilia*, the majority of them not having commonly occurring immunosuppression as risk factor.

## 5. Conclusions

It is evident from this study that ICU patients represent an emerging niche for *S. maltophilia* infections, and epidemiology of this primarily low virulence pathogen has to be more extensively upraised in the future in prospective multicenter studies. Although the significance of *S. maltophilia* in non-hematologic critically ill patients has not been extensively studied compared to that of *A. baumannii, S. maltophilia* has now been recognized as an emerging ICU pathogen and should raise a high index of suspicion [16,52]. Overall, this study highlights the importance of monitoring Stenotrophomonas infections in healthcare settings and implementing appropriate infection control measures to prevent and control the spread of multidrug-resistant strains.

## Figures and Tables

**Table 1:** Patients’ demographics and clinical characteristics.

Variable	Total Cohort *n* = 103	28 Day Survivors *n* = 47	28 Day Non-Survivors *n* = 56	*p*
**Demographics**				
Age years, median (IQR)	65.5 (54–73.3)	63.5 (47.8–70)	69 (58.3–76)	**0.010**
Gender male, *n* (%)	68 (66)	30 (63.8)	38 (67.9)	0.67
**Severity indices**				
APACHE II at ICU admission, mean (SD)	18.36 (7.22)	16.7 (7.17)	19.75 (7.03)	**0.032**
SOFA at ICU admission, mean (SD)	18.17 (6.95)	18.43 (7.83)	17.96 (6.19)	0.739
Sepsis at ICU admission	62 (60.2)	28/47 (59.6)	34/56 (60.7)	0.906
**Days in ICU, median (IQR)**	28 (16–53)	26 (17–53)	32 (15.3–53.8)	0.968
**Days in hospital, median (IQR)**	56 (30–89)	53 (30–85)	60 (31.3–101.3)	0.596
**Comorbidities by number**				0.339
None *n* (%)	28 (27.2)	16 (34)	12 (21.4)	0.152
One comorbidity *n* (%)	53 (51.5)	21 (44.7)	32 (57.1)	0.208
Two comorbidities *n* (%)	5 (4.8)	2 (4.3)	3 (5.4)	1.00
Three comorbidities *n* (%)	14 (13.6)	7 (14.9)	7 (12.5)	0.724
Four or more comorbidities *n* (%)	3 (2.9)	1 (2.1)	2 (3.6)	1.00
**Comorbidities by type**				0.157
Chronic renal disease *n* (%)	13 (12.6)	6 (12.8)	7 (12.5)	0.967
Diabetes mellitus *n* (%)	15 (14.6)	7 (14.9)	8 (14.3)	0.931
Chronic respiratory disease *n* (%)	9 (8.7)	5 (10.6)	4 (7.1)	0.779
Cardiovascular disease *n* (%)	25 (24.3)	11 (23.4)	14 (25)	0.851
Dyslipidemia/obesity *n* (%)	7 (6.8)	3 (6.4)	4 (7.1)	1.00
Hypertension *n* (%)	14 (13.6)	5 (10.6)	9 (16.1)	0.423
Hemato/oncologic malignancy *n* (%)	22 (21.3)	3 (6.4)	19 (33.4)	**0.003**
Solid Organ Transplantation *n* (%)	1 (1)	1 (2.1)	0 (0)	0.913
Pregnancy *n* (%)	1 (1)	1 (2.1)	0 (0)	0.913
Other *n* (%)	3 (2.9)	2 (4.3)	1 (1.8)	0.868
**Reason for ICU admission**				0.325
Medical cause	69 (67)	29 (62)	40 (71)	0.190
Surgical cause	34 (33)	18 (38)	16 (29)	0.170
**Main organ dysfunction**				0.190
Kidney	25 (25.3)	10 (21.3)	16 (28.5)	0.725
Respiratory	47 (45.6)	24 (51)	23 (41.1)	0.313
Cardiovascular	3 (2.9)	3 (6.4)	0 (0)	0.183
Liver	3 (2.9)	2 (4.3)	1 (1.8)	0.868
Hematologic	4 (3.9)	1 (2.1)	3 (5.4)	0.756
Multiple organs	16 (15.5)	4 (8.5)	12 (21.4)	**0.071**

ΙIQR: Interquartile Range; ICU: Intensive Care Unit; APACHE: Acute Physiological and Chronic Health Evaluation; SOFA: Sequential Organ Failure Assessment; SD: Standard Deviation.

**Table 2:** Clinical characteristics relating to the infection caused by *Stenotrophomonas maltophilia*.

Variable	Total Cohort *n* = 103	28 Day Survivors * n = 47	28 Day Non-Survivors * n = 56	*p*
**Severity indices**				
APACHE II at *S. maltophilia* isolation, median (IQR)	8 (5–15)	8 (4–14)	8.5 (6–15.5)	0.438
SOFA at *S. maltophilia* isolation, median (IQR)	7 (5–10)	7 (6–10)	8 (5–11)	0.950
Sepsis at *S. maltophilia* isolation	72 (69.9)	23 (68.1)	40 (71.4)	0.713
**Source of isolation**				0.374
Blood	4 (3.4)	1 (2.1)	3 (5.4)	
Tracheal aspirates	63 (61.2)	27 (57.4)	36 (64.3)	
Bronchoalveolar lavage fluid	9 (8.7)	5 (10.6)	4 (7.1)	
Sputum	1 (1)	0 (0)	1 (1.8)	
Pleural effusion	6 (5.8)	3 (6.4)	3 (5.4)	
Pharyngeal exudate	2 (1.9)	0 (0)	2 (3.6)	
Bile	1 (1)	0 (0)	1 (1.8)	
Surgical trauma	7 (6.8)	4 (8.5)	3 (5.4)	
Soft tissue/pus	1 (1)	1 (2.1)	0 (0)	
Peritoneal fluid	5 (4.8)	2 (4.2)	3 (5.4)	
Catheter	4 (3.4)	4 (8.5)	0 (0)	
**Type of infection**				0.264
Bloodstream infection	4 (3.4)	1 (2.1)	3 (5.4)	
VAP/HAP	75 (72.8)	32 (68.1)	43 (76.8)	
Pleural effusion/exudate	6 (5.8)	3 (6.4)	3 (5.4)	
Intraabdominal infection	6 (5.8)	2 (4.2)	4 (7.1)	
Surgical trauma infection	7 (6.8)	4 (8.5)	3 (5.4)	
Skin/soft tissue infection	1 (1)	1 (2.1)	0 (0)	
Catheter-related infection	4 (3.4)	4 (8.5)	0 (0)	
**Prior colonization with *S. maltophilia***				1.00
Yes	4 (3.4)	2 (4.3)	2 (3.6)	
**Type of antibiotics during *S. maltophilia* recovery**				0.449
Meropenem only	17 (16.5)	6 (12.8)	11 (19.6)	
Carbapenem combinations	24 (23.3)	12 (25.5)	12 (21.4)	
Piperacillin/tazobactam only	27 (26.2)	12 (25.5)	15 (26.8)	
Piperacillin/tazobactam combinations	13 (12.6)	9 (19.1)	4 (3.6)	
Clycopeptide or linezolid only	6 (5.8)	1 (2.1)	5 (8.9)	
Amoxicillin clavulanate or cephalosporin	5 (4.9)	2 (4.3)	3 (5.4)	
Quinolones only	4 (3.9)	1 (2.1)	3 (5.4)	
Quinolone/based combinations *	7 (6.8)	4 (8.5)	3 (5.4)	
**Prior carbapenem exposure**				0.775
Yes	41 (39.8)	18 (38.3)	23 (41.1)	
**Antibiotics administered for *S. maltophilia* infection**				0.112
Cotrimoxazole-based regimen	57 (55.3)	30 (63.8)	27 (48.2)	
Levofloxacin-based regimen	46 (44.7)	17 (36.2)	29 (51.8)	
**Presence of coinfection**				0.703
Yes	16 (15.5)	8 (17)	8 (14.3)	

* Combinations of a cephalosporin with a glycopeptide and quinolone; three patients in this subgroup were also administered colistin.

**Table 3:** Multivariate analysis for predictors of mortality.

	***p***	**OR**	**95% C.I.**	
Age	0.043	0.966	0.935	0.999
APACHE II upon ICU admission	0.438	0.974	0.912	1.041
Haematolo/oncologic disease	0.004	0.141	0.037	0.538
Multi-organ failure	0.148	0.388	0.108	1.399

OR: odds ratio; C.I.: confidence interval.

## Data Availability

All data can be made available upon reasonable requests and restrictions according to host institutions ethical committees.
